# National trends in advanced outpatient diagnostic imaging utilization: an analysis of the medical expenditure panel survey, 2000-2009

**DOI:** 10.1186/1471-2342-13-40

**Published:** 2013-11-26

**Authors:** Kathleen Lang, Huan Huang, David W Lee, Victoria Federico, Joseph Menzin

**Affiliations:** 1 Boston Health Economics, Inc., 20 Fox Road, Waltham, MA 02451, USA; 2 GE Healthcare, 9900 W. Innovation Drive, RP-2177, Wauwatosa, WI 53226, USA

**Keywords:** Diagnostic imaging, Utilization, Trend, Outpatient, MEPS

## Abstract

**Background:**

Concerns have been raised regarding growth in advanced diagnostic imaging use. This study evaluated trends in national outpatient MRI/CT utilization rates during 2000-2009 and factors associated with utilization.

**Methods:**

This retrospective database analysis used data on all respondents in the nationally representative U.S. Medical Expenditure Panel Survey (MEPS) during 2000-2009. Visits involving advanced diagnostic imaging were identified based on self-reported use of MRI or CT tests at emergency departments, office-based medical providers, and outpatient departments. The imaging utilization rate was defined as the number of outpatient visits with MRI/CT per 1,000 person-years. Results were weighted to create nationally representative estimates at the person-year level for each year and the pooled 10-year period. A multivariate logistic regression was estimated to identify predictors of imaging use.

**Results:**

A total of 319,246 person-years were included in the analysis. MRI/CT utilization rates increased from 64.3 to 109.1 per 1,000 person years from 2000 to 2009, with older persons, females and Medicare enrollees having higher rates of use. Growth in imaging slowed in recent years; the average annual decline in the imaging growth rate was larger than that for all outpatient services (4.7% vs. 0.9%). The percentage of respondents with MRI/CT use (6.7% during 2000-2009) also increased at a slower rate in later years and declined during 2007-2009. The average number of MRI/CT visits among imaging users was steady at about 1.5 visits during 2000-2009. Age, female gender, White race, HMO participation, and all payer types (vs. uninsured) were significant predictors of imaging use. Compared to 2005, years 2000-2003 were associated with a significantly lower likelihood of imaging use, while years 2004-2009 were not significantly associated, suggesting a slow-down in later years.

**Conclusions:**

Growth in advanced imaging utilization appears to have slowed in recent years, a finding of potential interest to policy-makers and payers.

## Background

It has been well documented that use of diagnostic imaging technology grew rapidly in the early 2000s [1-5]. The rate of growth in imaging use was substantial, with a 70% cumulative increase in utilization between 2000 and 2007 among Medicare beneficiaries, compared to a less than 40% increase for all physician services [2]. Between 2000 and 2006 annual Medicare spending on magnetic resonance imaging (MRI), computed tomography (CT), and positron emission tomography (PET) scans rose from $3.6 billion to $7.6 billion (approximately 17% per year) [2].

Despite high growth rates observed in the early 2000s, recently there have been signs that rates of imaging utilization began to slow around 2005 [6-9]. It has been shown that the compound annual growth rate of CT use among Medicare beneficiaries has dropped substantially when comparing 2000-2006 and 2007-2009 and has fallen even more drastically for MRI [6,9].

While these results suggest a slowing trend in the growth of imaging utilization among Medicare beneficiaries, [7-10] there are limited recent data examining similar trends across all payers. Lee and Levy reported limited data showing a general slowdown in the use of CT and MRI among commercial payers, and Bhargavan and colleagues evaluated imaging utilization at the national level, but only included data as recent as 2001 [1,6]. Our study aimed to: (1) evaluate whether the slowing utilization observed in the Medicare population also occurred among other populations (e.g., patients with other insurance coverage, different demographic groups), (2) assess whether the observed trends in utilization continued into 2009, and (3) identify factors associated with diagnostic imaging utilization.

## Methods

### Data source

This retrospective database analysis used data from the U.S. Medical Expenditure Panel Survey (MEPS), a continuous, ongoing dataset of large-scale surveys of families and individuals, their medical providers, and insurers across the U.S. that gather data on the frequency of use and costs of specific health services. Information is collected from a nationally representative sample of the U.S. civilian noninstitutionalized population using a complex, multi-stage survey design.

The 10-year MEPS sample (2000-2009) used in this study includes data on 319,246 person-years. The MEPS Full Year Consolidated (FYC) Files, Emergency Room (ER) Visits Files, Office-based Medical Provider Visits Files, and Outpatient Department Visits Files were used. Free-standing imaging centers are captured in the Office-based Medical Provider Visits Files.

The FYC File summarizes all of the person-level variables in the same year into one file, including demographics, health insurance, and a person weight which is used to create national estimates based on the sample collected in MEPS. Relevant variables in the encounter-level files (e.g., ER visits) include the visit date and whether there was imaging use during the visit [11]. All data files used in these analyses are publicly available for download on the MEPS website [12].

### Patient selection

All MEPS respondents between 2000 and 2009 were included in the analysis for all years in which they had a positive person weight. Similar to other published studies, unique combinations of patient ID and year were used to identify person-year observations (i.e., the same person in two consecutive years was treated as two different people) [13,14].

### Study measures

Outpatient visits from any of the outpatient files (i.e., emergency department [ED], office-based medical provider, and outpatient department visits) involving MRI/CT were identified based on an affirmative answer to: “Did the person have an MRI or CT during this visit?” As we were unable to discern the number of imaging procedures the person had at the visit, the imaging utilization rate was defined as the number of visits with MRI/CT per 1,000 persons, rather than the number of procedures per 1,000 persons. Similarly, the overall outpatient visit rate, used as a reference point, was defined as the total number of all visits per 1,000 persons. The proportion of respondents who had at least one outpatient visit with MRI/CT and the proportion of outpatient visits with MRI/CT were also assessed.

Among those who had at least one visit for an MRI/CT during a year, the average number of outpatient visits with an MRI/CT for that year was also evaluated. The average annual growth rate in imaging utilization was calculated as the average of the annual growth rates for all individual years during a given time period [15]. The average annual decrease in the growth rate was calculated as: (annual growth rate in the last year–annual growth rate in the first year)/number of years.

### Data analysis

Descriptive analyses of all study measures were performed. Data from all years were pooled into an overall study database, and all study measures were analyzed for each year and for the 10-year period. Results were weighted to the national level using longitudinal person weights. These survey weights are provided by MEPS to adjust for unequal probabilities of selection into the survey, as well as for survey nonresponse and attrition. Analyses were stratified by patient demographics, payer type, and managed care participation.

Each respondent was grouped into one of the following payer categories: Medicare only, Medicaid only, Private only, Medicare + Medicaid, Medicare + Private, Other Public (TRICARE, State covered, other public), Uninsured, and Other. MEPS provides information on payer status for each month; payer type was assigned as the payer of longest duration during the year. Those who had the same duration of coverage with multiple insurance types were classified as having “Other” coverage.

Managed care participation was defined as whether a person was covered under a managed care plan (public or private), including plans defined as an HMO, gatekeeper plans, or plans with a doctor list [16].

Age-and gender-adjusted imaging and outpatient visit rates were evaluated, in addition to the unadjusted rates, based on the direct standardization method, using population sizes obtained from US Census Bureau, American Community Survey 2009 [17,18]. Adjusted rates could not be calculated for stratified analyses by payer type and managed care participation due to a lack of data by payer type in the American Community Survey.

A multivariate logistic regression was estimated to identify factors associated with a higher likelihood of imaging use. Predictors included age, sex, race, region, urban/rural, managed care participation, payer type, and year (with 2005 as the reference category). We chose 2005 as the reference year because other studies have indicated that CT and MRI use were lower during the period 2005-2008, compared to 1998-2005 [6,8]. Three supplementary logistic regressions with alternative specifications for the year variable were estimated to test whether growth in imaging was slower after 2005 (Details regarding the methods and results of these supplementary regressions are provided in the Appendix). Statistical significance was defined as a *P*-value less than 0.05. SAS software (Version 9.1, SAS Institute, Cary, NC) was used for all analyses.

## Results

### Population characteristics

Approximately 25% of the sample was less than 18 years old, 38% between 18 and 44, 25% between 45 and 64 and 12% aged 65 and older over the entire 10-year period. Males comprised 49% of the population and over 80% were from urban areas. Demographics were generally similar in each year over the 10-year period. The gender distribution remained constant, as did the age distribution, with a slight trend of increasing proportions falling into the older age groups. Nearly two-thirds of the population was White, with a slightly lower prevalence in later years (71% in 2000 and 65% in 2009).

The distribution of the population among payer types remained fairly steady over the 10-year period. There was a small decrease in the percent of people with private insurance (59.2% in 2000 and 52.4% in 2009), which corresponded with increases in the proportion of patients with Medicaid (7.4% in 2000 and 11.4% in 2009) and without health insurance (16.8% in 2000 and 17.8% in 2009). There was also an increase in the percent with managed care insurance over the 10-year period (47.4% in 2000 vs. 55.4% in 2009).

### Imaging utilization rates

**Figure 1:**
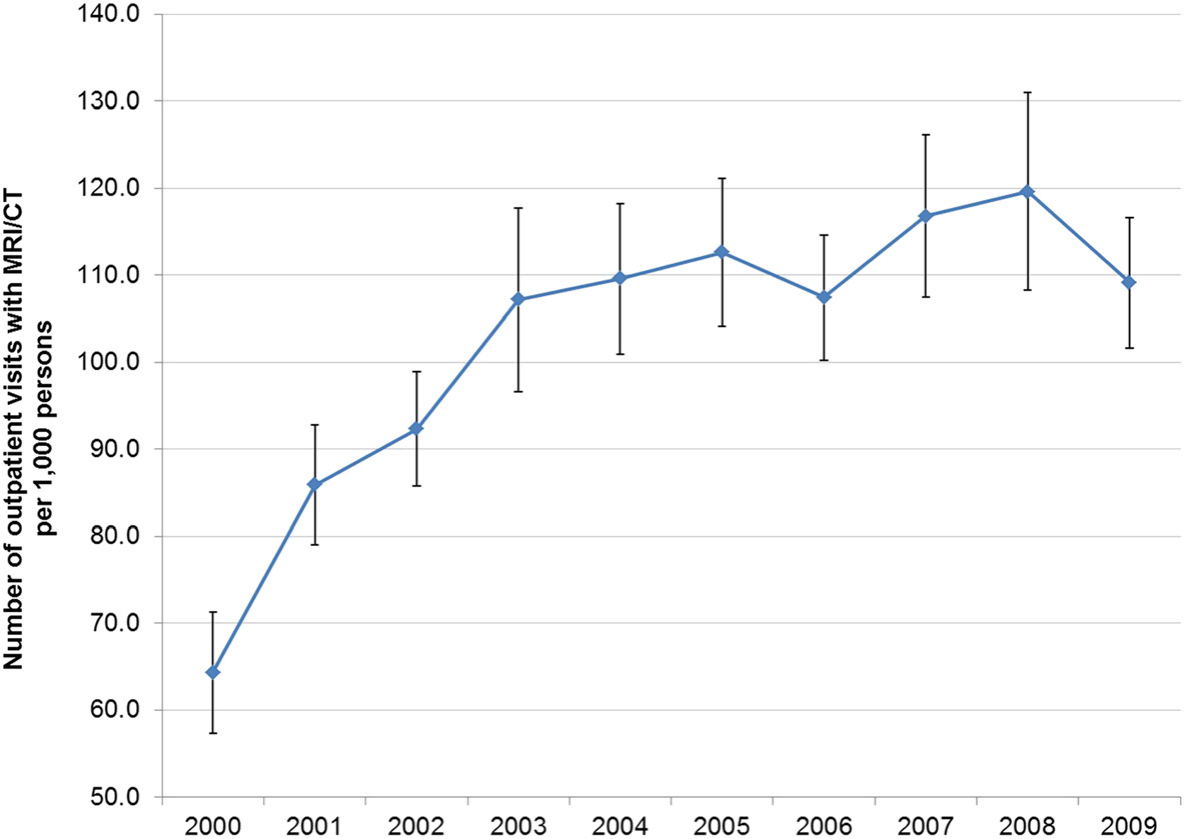
Imaging utilization rate, by year.

Overall, MRI/CT utilization rates increased from 64.3 to 119.6 per 1,000 persons from year 2000 to 2008 and then declined to 109.1 per 1,000 persons in 2009. Utilization rates increased at a rate of just under 15% in the first five years of the time period and then at an annual average growth rate of almost 0% for 2005-2009 (Table 1, Figure 1). This trend was observed across almost all patient groups (i.e., defined by age, gender, race, and region) and payor types. Age-and gender-adjusted rates (results not shown) were similar to unadjusted rates, reflecting the minimal changes in patient demographics over the 10-year window.

**Table 1:** Imaging utilization rate (number of outpatient visits with MRI/CT per 1,000 persons), by year and patient characteristics

	**2000**	**2001**	**2002**	**2003**	**2004**	**2005**	**2006**	**2007**	**2008**	**2009**	2000-2009*
Unweighted N	23,839	32,122	37,418	32,681	32,737	32,320	32,577	29,370	31,262	34,920	319,246
Imaging utilization rate	64.3	85.9	92.3	107.2	109.6	112.6	107.4	116.8	119.6	109.1	102.9
Age											
<18	14.3	20.4	24.2	29.3	20.3	27.1	21.4	24.1	27.4	26.8	23.6
18-44	44.5	74.7	67.1	62.9	87.0	81.1	80.5	72.7	73.4	74.8	71.9
45-64	97.5	121.9	141.3	186.0	161.5	170.6	164.1	171.8	185.8	172.5	159.1
65+	160.9	182.6	219.3	244.0	253.8	261.4	241.4	309.3	282.8	234.3	240.4
Gender											
Male	54.7	78.1	80.3	92.2	93.5	98.6	92.6	106.3	98.8	99.4	89.8
Female	73.5	93.3	103.8	121.6	125.0	126.1	121.7	126.9	139.6	118.6	115.4
Race/ethnicity											
White	73.5	95.9	108.5	126.3	128.6	135.3	127.3	137.9	146.6	133.7	121.4
Non-white	41.7	62.9	58.3	67.3	71.2	67.8	69.1	76.7	68.8	63.7	65.3
Census region											
Northeast	72.3	109.2	97.1	108.7	129.3	120.7	128.8	140.1	135.3	112.7	115.6
South	64.6	81.5	93.1	114.6	112.0	116.4	109.9	111.2	102.2	110.3	102.1
Midwest	57.3	88.1	96.2	95.0	109.7	119.9	105.2	118.6	131.4	108.7	103.2
West	56.2	66.5	83.0	102.2	87.7	91.8	86.5	101.6	117.3	104.6	90.3
Rural/urban residence											
MSA	61.0	87.1	89.0	106.3	106.7	111.9	105.3	116.0	118.7	110.2	101.8
Non-MSA	68.9	74.5	106.4	105.8	120.6	113.7	114.9	114.7	114.5	102.7	103.4
Payer type											
Medicare	131.4	195.2	187.0	238.7	241.3	255.6	261.8	276.0	303.8	223.1	234.2
Medicaid	32.2	62.1	65.8	60.3	58.3	78.9	67.8	58.1	69.1	62.4	62.5
Private	52.8	78.0	84.0	91.2	96.7	97.8	95.1	98.7	105.4	96.9	89.6
Medicare + Medicaid	183.6	124.4	248.5	194.9	269.5	227.5	172.0	240.9	219.0	357.5	226.8
Medicare + Private	180.5	188.0	237.9	253.2	265.0	295.9	250.6	364.0	300.6	253.3	257.8
Other public**	243.0	235.1	78.4	100.5	98.2	122.7	104.7	133.1	123.4	149.3	131.4
Uninsured	32.9	37.2	30.8	74.3	61.1	54.8	58.1	60.6	48.9	56.2	51.8
Other	91.2	96.8	134.4	175.1	165.5	160.2	165.1	141.0	205.9	164.3	152.5
Managed care status											
Managed care	59.0	80.2	86.4	97.3	103.1	107.9	108.3	118.6	113.4	114.9	99.9
Non-managed care	69.1	93.8	100.7	121.0	118.5	119.0	106.2	114.4	127.2	102.0	106.8

Source: Medical Expenditure Panel Survey 2000-2009.

MSA: Metropolitan Statistical Area.

Note: Outpatient visits include outpatient, office-based, and ER visits.

Note: Weighted results are reported to reflect US noninstitutionalized population.

Note: 2000-2009 results represent weighted, pooled data from each year from 2000-2009.

*2000-2009 estimates were calculated on a pooled sample of imaging visits over all data years.

**Other public includes Tricare, State covered insurance and other public insurance.

**Figure 2:**
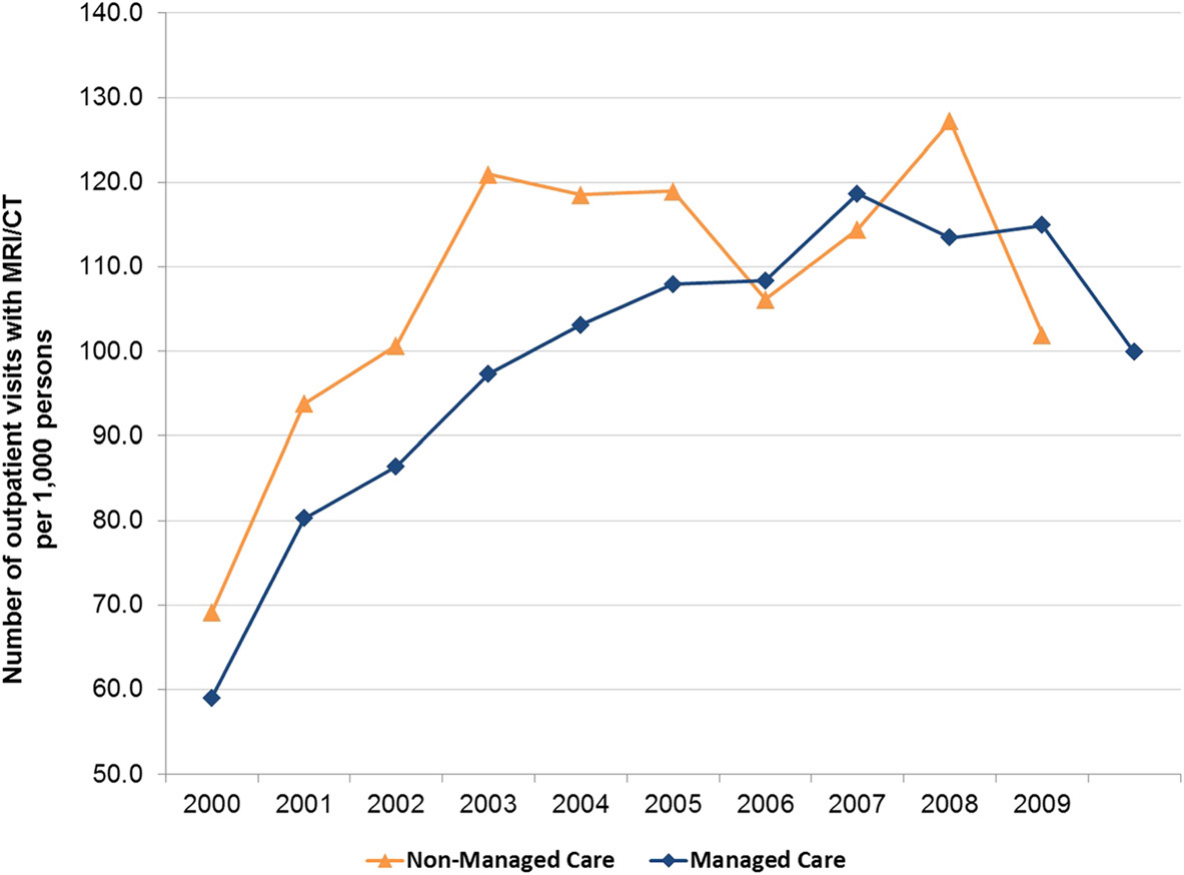
Imaging utilization rate, by year and managed care participation.

Older people tended to have higher rates of imaging use, with average MRI/CT utilization rates of approximately 24, 72, 159, and 240 per 1,000 persons for ages <18, 18-44, 45-54 and 65+ years, respectively. Females had higher rates of imaging use than males (115 vs. 90 per 1,000 persons during 2000-2009). The uninsured and those with only Medicaid coverage had similar rates of imaging use that were the lowest rates observed over 2000-2009 across all payer types (52 per 1,000 persons for the uninsured and 63 per 1,000 persons for those with Medicaid only). In contrast, those with any Medicare coverage had the highest rates of utilization (ranging from 227 to 258 per 1,000 persons for these payer groups over the 10-year period). In the earlier years (2000-2005), imaging utilization among those with non-managed care insurance was consistently higher than among those with a managed care plan. However, in the most recent years (2006-2009), the utilization rates by managed care participation appeared to converge (Figure 2).

The average decrease in the annual growth rate for MRI/CT use between 2000 and 2009 (4.7%) was larger than the average decrease in the general outpatient visit growth rate (0.9%). In addition, the percent of outpatient visits involving an MRI/CT increased from 2000 (1.3%) to 2008 (2.1%), then decreased in 2009 to 1.9%. The average decrease in the annual growth rate for the percent of outpatient visits involving MRI/CT (3.5%) was also larger than the average decrease in the general outpatient visit growth rate. These results suggest a larger slowdown in MRI/CT growth than in overall outpatient services growth.

The percentage of patients who had at least one visit involving MRI/CT use increased at a slower rate in more recent years and decreased between 2007 and 2009. The percentage of patients with at least one visit with imaging use was highest among those aged 65+ years, at approximately 15.0% over 2000-2009. Females were more likely than males to use MRI/CT: 7.5% vs. 5.8% respectively over the 10-year period. The uninsured and people with Medicaid were least likely to use MRI/CT services (3.4% and 3.9% during 2000-2009, respectively), while those with any Medicare coverage were most likely, ranging from 13.1% to 16.3%. Managed care plan holders were slightly less likely to receive an imaging test (6.6% vs. 6.9% during 2000-2009), compared to non-managed care plan holders. The average number of visits with an MRI/CT performed, among those people who had at least 1 imaging visit, was steady (ranging from 1.4 to 1.6) during the 2000-2009 period for all stratifications.

### Predictors of imaging utilization

Results from a multivariate logistic regression indicated that increasing age, female gender, White race, living in an urban area, having managed care insurance, and having any insurance were significantly positively associated with having an outpatient visit involving MRI/CT (Table 2). Compared to 2005, years 2000-2003 were associated with a significantly lower likelihood of having MRI/CT visits, while years 2004 and 2006 through 2009 were not significant predictors of use, suggesting a slow-down in advanced imaging utilization in later years.

**Table 2:** Factors associated with likelihood of having an outpatient MRI/CT visit

**Covariate**	**OR estimate**	**95% confidence limits**	
Managed care vs. non-managed care	**1.066**	**1.015**	**1.120**
Payer type (vs. uninsured)			
Medicare	**2.749**	**2.437**	**3.102**
Medicaid	**1.987**	**1.799**	**2.195**
Private	**1.619**	**1.505**	**1.741**
Medicare + Medicaid	**3.076**	**2.708**	**3.493**
Medicare + Private	**3.027**	**2.678**	**3.422**
Other public*	**2.194**	**1.818**	**2.647**
Other	**2.375**	**2.153**	**2.620**
Year (vs. 2005)			
2000	**0.579**	**0.523**	**0.642**
2001	**0.737**	**0.678**	**0.801**
2002	**0.865**	**0.798**	**0.937**
2003	**0.890**	**0.828**	**0.956**
2004	0.950	0.882	1.025
2006	0.987	0.915	1.064
2007	1.043	0.971	1.120
2008	1.013	0.936	1.097
2009	0.969	0.894	1.051
Age (vs. <18 years)			
18-44 years	**2.954**	**2.746**	**3.178**
45-64 years	**5.514**	**5.126**	**5.931**
65+ years	**5.016**	**4.469**	**5.630**
Male (vs. female)	**0.820**	**0.790**	**0.850**
White (vs. non-white)	**1.598**	**1.526**	**1.674**
Census region (vs. Northeast)			
South	0.985	0.924	1.050
Midwest	0.970	0.904	1.041
West	**0.843**	**0.780**	**0.911**
Urban (vs. rural)	**1.056**	**1.000**	**1.114**

Source: Medical Expenditure Panel Survey 2000-2009.

Note: Outpatient visits include outpatient, office-based, and ER visits.

*Other public includes Tricare, State covered insurance and other public insurance.

Note: Bold indicates statistical significance (P-Value < 0.05).

Note: Regression is based on weighted results.

**Figure 3:**
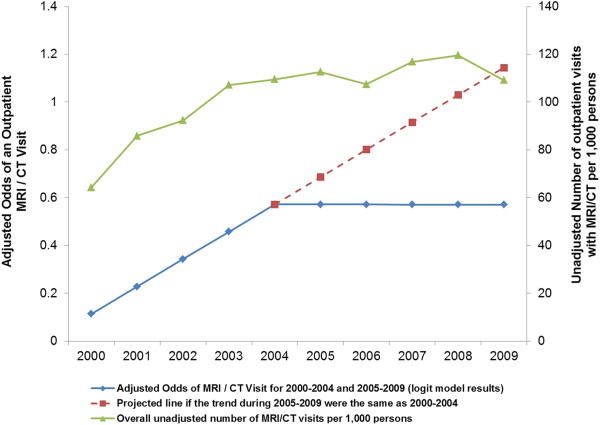
Multivariate logistic regression results of odds of an outpatient MRI/CT visit during 2000-2004 and 2005-2009 versus unadjusted overall number of MRI/CT visits per 1,000 persons.

In three supplementary logistic regressions involving alternative specifications for the year variable, we confirmed that imaging rates increased significantly in years 2000-2004 and then stabilized during the 2005–2009 period (see Appendix). A schematic illustration of these results is shown in Figure 3.

## Discussion

### Summary & implications

This study evaluated nationally representative trends in outpatient imaging utilization over the last decade stratified by patient age, gender, region of residence, and insurance type, and identified key factors associated with imaging utilization. Results suggested an average growth rate in imaging utilization of 15% over the first five years of the decade (2000-2004), then leveling off to an approximately 0% growth rate between 2005-2009. After controlling for patient age, female gender, race, and type of insurance coverage, the years 2000-2003 were statistically significantly associated with higher likelihood of imaging use vs. the year 2005, while years 2004 and 2006-2009 were not significant predictors of imaging utilization, therefore confirming the unadjusted results of a slowdown in imaging use in recent years.

Our analysis suggests that utilization of CT and MRI has slowed not only among Medicare beneficiaries, but also among those with other types of insurance. This slowdown outpaced declines in the growth rate for outpatient services overall, indicating that the stabilizing trend of imaging use can be attributed to reasons other than just declines in growth in all-cause outpatient visits per year. These findings suggest that the greater observed slowdown in imaging use vs. outpatient visits overall may not merely be due to recent economic trends. Given that patterns of imaging use were similar among patients with Medicaid coverage and patients without any insurance coverage, and given the expectation that over half of the currently uninsured will qualify for Medicaid under the 2010 Patient Protection and Affordable Care Act, our findings suggest that health reform may not cause a drastic increase in advanced imaging utilization as the number of insured increases [19].

Other potential reasons for this observed slowdown in imaging use are suggested by Levin et al. and Sharpe et al. in their respective analyses of trends of CT and MRI use [20,21]. First, there is a greater awareness among both physicians and patients of radiation concerns associated with imaging use. Radiologists have been working to educate healthcare professionals and improve patient awareness concerning radiation exposure through educational campaigns, such as the introduction of the Image Wisely campaign by the American College of Radiology (ACR) and the Radiological Society of North America (RSNA) in 2010 [22]. Second, cost concerns may cause physicians to pay more attention to the ACR appropriateness criteria for imaging use [23].

### Comparison to literature

The rates of advanced diagnostic imaging utilization observed in this study among Medicare patients are lower than those reported in studies using the Medicare Physician Supplier files. Levin and colleagues report a rate of outpatient CT and MRI use of 437 per 1,000 Medicare beneficiaries in 2008, compared to our results ranging from 219-304 outpatient visits with a MRI/CT per person among people with Medicare only, Medicare + Private insurance or Medicare + Medicaid in 2008 [8]. This discrepancy was also observed in Bhargavan and colleagues’ 2005 study, which examined both the Medicare Physician Supplier files and MEPS data to assess utilization. They report an ambulatory MR/CT utilization rate of 151 procedures per 1,000 person 65 years or older using 1999 MEPS data and a rate of 341 procedures per 1,000 Medicare beneficiaries in 2001 using the Physician Supplier files [1]. These differences may be due to the fact that analyses of administrative claims from the Medicare Physician Supplier files are based on Current Procedural Terminology (CPT) codes for imaging procedures, whereas analyses using MEPS are based on self-report. However, MEPS administrators do make an attempt to verify respondent reported information. Additionally, due to the nature of the question “Did the person have an MRI or CT during this visit?”, we cannot capture multiple procedures occurring during the same visit, which is why we report utilization rates as ‘number of visits with MRI/CT’, rather than MRI/CT procedures.

While the absolute utilization rates reported here may differ from other studies using administrative claims, the MRI/CT use question in MEPS was asked consistently over the 10-year period; therefore, our observations related to the trend of decelerating growth in advanced diagnostic imaging use are consistent with other studies among Medicare populations. Similar to our findings, a 2012 study of outpatient (i.e., office and hospital outpatient department) imaging use among Medicare beneficiaries observed a distinct flattening of trend lines in CT and MRI use in outpatient settings between 2005 and 2006 [6,9]. This study reported the compound annual growth rate for CT use fell from 17.5% for office visits and 8.6% for hospital outpatient departments between 2000 and 2006 to 2.1% and 0.5%, respectively, between 2007 and 2009. Similarly, MRI utilization rates fell from 14.9% and 11.3% for office and hospital outpatient departments, between 2000 and 2006 to −1.1% and 1.0% between 2007 and 2009 [9]. Other analyses of Medicare Part B data have reported that the rate of growth for advanced imaging technologies including CT, MRI, and PET had fallen between 2006-2008 to as low as 1-2% [7,24]. These trends are in line with our results for outpatient MRI/CT utilization rates among those with Medicare coverage, where the average annual growth rate was 11.1% during 2000-2005 and 3.7% during 2005-2008 (−0.7% over 2005-2009). Our results showed that the annual growth rate in national outpatient MRI/CT utilization followed the same pattern as observed in the Medicare population: 12.4% over 2000-2005 falling to 2.2% over 2005-2008 (to −0.6% for 2005-2009).

Previous studies have reported inconsistent findings regarding the relationship between managed care membership and use of imaging services [4,25,26]. In an analysis of electronic medical record data from HMO enrollees in six integrated health systems, results suggested that both CT and MRI use increased between 1996 and 2010 [26]. Conversely, results from a nationwide “census” of MRI sites in 1993, 1997, and 1999 suggested that metropolitan statistical areas with a higher HMO market share had statistically significantly lower adoption and use of MRI scanners compared to areas with lower HMO prevalence [16]. Our study suggested, in descriptive analyses, that patients enrolled in managed care plans were slightly less likely (6.6% vs. 6.9%) to utilize MRI/CT services, but the utilization rate appeared to converge in later years. Furthermore, in multivariate regression, patients in managed care plans were found to be significantly more likely to use imaging services after controlling for other characteristics (payer type, year, age, gender, race, region and urban/rural status). The uncertainty surrounding the relationship between managed care plan membership and imaging use, and a lack of published data on this potential association, raise important questions about whether financial incentives play a role in imaging use. This relationship merits further research given the Federal government’s historical promotion of managed care and reduced imaging reimbursement as a means to reduce costs [27-29].

In contrast to our findings that females have a significantly higher odds of MRI/CT use versus males, results from a prior retrospective study of determinants of imaging utilization in primary care found that women did not have a statistically significant greater odds of imaging use, [30]. However, our results are not directly comparable to those of Sistrom et al. as their study grouped several types of imaging use into one study measure, including MRI, CT, nuclear medicine, positron emission tomography (PET), x-ray, and ultrasound. Unadjusted results from their analyses do show a numerically higher rate of imaging visits among females (58% of all visits). One possible reason for the significance observed in our study may be the greater utilization of medical care services in general among females. According to a recent estimate by the Agency for Healthcare Research and Quality (AHRQ), men were 24% less likely than females to visit a doctor within the past year [31].

### Limitations

As this study is a retrospective analysis of household survey data, it is subject to limitations of the accuracy of respondent recall and the potential for reporting bias. In addition, imaging use in the inpatient setting was not captured in this analysis, and therefore these results may not be generalizable to imaging use outside of the outpatient setting.

This study only analyzed outpatient visits for MRI/CT, excluding inpatient imaging use, similar to approaches in prior retrospective analyses of imaging use [1,8,9]. Therefore, the findings presented in the current study may not be generalizable to inpatient settings. However, results from a recent analysis of Medicare data suggest that rates of CT use declined between 2009 and 2010 in both the inpatient and outpatient settings [20]. Additionally, this analysis did not report trends in MRI/CT use in the ED vs. other outpatient visits separately. Recent literature has suggested that rates of CT use in the ED are rose dramatically between the mid-1990s and 2007 [32-34]. One analysis of National Hospital Ambulatory Medical Care Survey (NHAMCS) showed a 4.9 fold increase in the percentage of all ER visits with CT use between 1995 and 2007, [34] with this finding confirmed in a similar study of NHAMCS data over 1996 to 2007 [32]. Future analysis of trends in ED-specific imaging are warranted, particularly to examine whether there is evidence of a slowdown in CT use post-2007, and to examine MRI/CT use jointly.

Another limitation of these analyses is the documented under-reporting of ED visits in MEPS data. In a comparison of the total number of ED visits reported in the National Hospital Ambulatory Medical Care Survey (NHAMCS) versus MEPS data, results showed a total of 130 million ED visits in NHAMCS versus 49 million in MEPS [35]. Potential reasons cited for the under-counting of ED encounters in MEPS are the undersampling of patients admitted to an inpatient setting from the ED, the exclusion of institutionalized patients, and underreporting of patients with Medicaid coverage [35].

Finally there seemed to be an anomaly in our results for 2006 that did not follow the general observed trend in the imaging utilization rate (number of outpatient visits with MRI/CT per 1,000 person). Overall, the imaging utilization rate declined slightly in 2006 and then increased in 2007 and 2008, but then declined again in 2009. In the Medicare-only population, the imaging utilization rate increased steadily from year 2000 to year 2008, without a decline in year 2006, which was consistent with other published studies among Medicare beneficiaries [7-9].

## Conclusions

These results provide valuable insights into how patterns of outpatient advanced imaging use have changed over time. This more comprehensive understanding of recent trends and patterns in imaging utilization can inform policy makers and payers as they develop future imaging-related policies.

## Appendix: supplementary regressions

Three supplementary logistic regressions including the same predictors as the original regression and alternative specifications for the year variable were estimated to test whether growth in imaging was slower after 2005.

The first supplementary regression (shown in Table 3), included two continuous year variables, one with values 1 through 10 for years 2000-2009 and the other with values 1 through 5 for years 2005-2009 (with the value zero for other years). For years 2000-2004, the estimated slope of the best fit line showing the influence of year on the likelihood of imaging use is based on the coefficient for the first year variable, while the slope estimate for 2005-2009 is the combination of the two year variable coefficients. Results suggest that imaging rates increased significantly in the early years and then stabilized after 2005 (since the sum of the coefficients is close to zero). The significance of the 2005-2009 year variable suggests that growth in imaging was significantly different for the two periods.

**Table 3:** Multivariate logistic regression model predicting the likelihood of having an outpatient MRI/CT visit using 2000-2009 data

**Covariate**	**Parameter estimate**	***P*-Value**
Managed care vs. non-managed care	**0.068**	**0.007**
Payer type (vs. uninsured)		
Medicare	**1.010**	**<.0001**
Medicaid	**0.685**	**<.0001**
Private	**0.480**	**<.0001**
Medicare + Medicaid	**1.122**	**<.0001**
Medicare + Private	**1.106**	**<.0001**
Other public*	**0.787**	**<.0001**
Other	**0.865**	**<.0001**
Year**		
Year A	**0.114**	**<.0001**
Year B	**−0.115**	**<.0001**
Age (vs. <18 years)		
18-44 years	**1.083**	**<.0001**
45-64 years	**1.708**	**<.0001**
65+ years	**1.614**	**<.0001**
Male (vs. female)	**−0.199**	**<.0001**
White (vs. non-white)	**0.468**	**<.0001**
Census region (vs. Northeast)		
South	−0.015	0.647
Midwest	−0.030	0.400
West	**−0.171**	**<.0001**
Urban (vs. rural)	0.053	0.054

Source: Medical Expenditure Panel Survey 2000-2009.

Note: Outpatient visits include outpatient, office-based, and ER visits.

Note: Bold indicates statistical significance (P-Value < 0.05).

Note: Regression is based on weighted results.

*Other public includes Tricare, State covered insurance and other public insurance.

**The parameter estimate for Year A represents the best fit slope for 2000-2004 after adjusting for other factors. The sum of parameter estimates for Year A and Year B (−0.0002, P-value 0.97) represent the best fit slope for 2005-2009. Year A and Year B were coded as follows:

2000: Year A (1); Year B (0).

2001: Year A (2); Year B (0).

2002: Year A (3); Year B (0).

2003: Year A (4); Year B (0).

2004: Year A (5); Year B (0).

2005: Year A (6); Year B (1).

2006: Year A (7); Year B (2).

2007: Year A (8); Year B (3).

2008: Year A (9); Year B (4).

2009: Year A (10); Year B (5).

In the second regression including data from only 2005-2009 and one continuous year variable, the year variable was not significant, suggesting no growth in imaging during this period (Table 4). The third regression including data from 2000-2004 and one continuous year variable produced a coefficient very similar to that estimated for years 2000-2004 in the first regression, confirming significant growth during the early years (Table 5).

**Table 4:** Multivariate logistic regression model predicting the likelihood of having an outpatient MRI/CT visit using 2005-2009 data

**Covariate**	**Parameter estimate**	***P*-Value**
Managed care vs. non-managed care	**0.0813**	**0.0075**
Payer type (vs. uninsured)		
Medicare	**1.0348**	**<.0001**
Medicaid	**0.6906**	**<.0001**
Private	**0.4543**	**<.0001**
Medicare + Medicaid	**1.0667**	**<.0001**
Medicare + Private	**1.1154**	**<.0001**
Other public*	**0.7998**	**<.0001**
Other	**0.8215**	**<.0001**
Year**	−0.00434	0.6466
Age (vs. <18 years)		
18-44 years	**1.0995**	**<.0001**
45-64 years	**1.7388**	**<.0001**
65+ years	**1.6151**	**<.0001**
Male (vs. female)	**−0.2048**	**<.0001**
White (vs. non-white)	**0.4462**	**<.0001**
Census region (vs. Northeast)		
South	−0.0119	0.7565
Midwest	−0.0421	0.3601
West	**−0.1758**	**0.0002**
Urban (vs. rural)	**0.089**	**0.032**

Source: Medical Expenditure Panel Survey 2005-2009.

Note: Outpatient visits include outpatient, office-based, and ER visits.

Note: Bold indicates statistical significance (P-Value < 0.05).

Note: Regression is based on weighted results.

*Other public includes Tricare, State covered insurance and other public insurance.

**Year was coded as follows: year 2005 = 1, 2006 = 2, 2007 = 3, 2008 = 4, 2009 = 5.

**Table 5:** Multivariate logistic regression model predicting the likelihood of having an outpatient MRI/CT visit using 2000-2004 data

**Covariate**	**Parameter estimate**	***P*-Value**
Managed care vs. non-managed care	**0.044**	**0.237**
Payer type (vs. uninsured)		
Medicare	**0.966**	**<.0001**
Medicaid	**0.681**	**<.0001**
Private	**0.518**	**<.0001**
Medicare + Medicaid	**1.194**	**<.0001**
Medicare + Private	**1.093**	**<.0001**
Other public*	**0.761**	**<.0001**
Other	**0.924**	**<.0001**
Year**	**0.113**	**<.0001**
Age (vs. <18 years)		
18-44 years	**1.064**	**<.0001**
45-64 years	**1.669**	**<.0001**
65+ years	**1.616**	**<.0001**
Male (vs. female)	**−0.191**	**<.0001**
White (vs. non-white)	**0.500**	**<.0001**
Census region (vs. Northeast)		
South	−0.019	0.691
Midwest	−0.015	0.752
West	**−0.166**	**0.003**
Urban (vs. rural)	0.014	0.661

Source: Medical Expenditure Panel Survey 2000-2004.

Note: Outpatient visits include outpatient, office-based, and ER visits.

Note: Bold indicates statistical significance (P-Value < 0.05).

Note: Regression is based on weighted results.

*Other public includes Tricare, State covered insurance and other public insurance.

**Year was coded as follows: year 2000 = 1, 2001 = 2, 2002 = 3, 2003 = 4, 2004 = 5.

A schematic illustration of these results is shown in Figure 3 of the full text.

## Competing interests

This study was sponsored by GE Healthcare. Dr. Lang, Dr. Huang, Ms. Federico and Dr. Menzin declare that they have no competing interests. Dr. Lee is an employee of the sponsor (GE Healthcare, Wauwatosa, WI).

## Authors’ contributions

KL, JM, DWL, and HH designed the research methods. KL, HH, and VF collected and analyzed the data. All authors contributed to data interpretation, made substantive contributions to the manuscript, and had final approval of the article.

## Pre-publication history

The pre-publication history for this paper can be accessed here:

http://www.biomedcentral.com/1471-2342/13/40/prepub
